# Modified Anti-PstS1 Bi-specific antibodies unlock potent protection against tuberculosis

**DOI:** 10.1371/journal.ppat.1014133

**Published:** 2026-05-27

**Authors:** Akanksha, Rania Bouzeyen, Avia Watson, Gil Wiseglass, Nyaradzai Sithole, Lilach Abramovitz, Noam Ben-Shalom, Rotem Rubinstein, Babak Javid, Natalia T. Freund

**Affiliations:** 1 Gray School of Medical Sciences, Gray Faculty of Medical & Health Sciences, Tel Aviv University, Tel Aviv-Yafo, Israel; 2 Division of Experimental Medicine, University of California San Francisco, San Francisco, California, United States of America; 3 Department of Biochemistry & Molecular Biology, School of Neurobiology, Biochemistry & Biophysics, The George S. Wise Faculty of Life Sciences, Tel Aviv University, Tel Aviv-Yafo, Israel; 4 Department of Medicine, University of Cambridge, Cambridge, United Kingdom; New Jersey Medical School, UNITED STATES OF AMERICA

## Abstract

The role of antibodies in the host response against *Mycobacterium tuberculosis* (*M. tb*) bacteria is still poorly understood. We previously isolated two monoclonal antibodies (mAbs), p4-36 and p4-163, from an *M. tb* infected donor that target two non-overlapping epitopes on PstS1, a subunit of the *M. tb* phosphate transporter. Although these antibodies reduced lung bacterial burden in mice (30–40% reduction in CFU), their efficacy remained modest for therapeutic application. Here, we employed a rational antibody engineering approach to further enhance their anti-*M. tb* potency. Affinity maturation of p4-163 yielded p4-163LR, a variant with superior binding to PstS1 and improved recognition of live, attenuated *M. tb*. Surprisingly, p4-163LR alone did not confer enhanced protection against virulent *M. tb in vivo*. However, the generation of a bispecific antibody combining p4-36 and p4-163LR (Bi-S 36/163LR) significantly improved bacterial binding and antibody-dependent cellular phagocytosis (ADCP). Notably, prophylactic administration of Bi-S 36/163LR led to a ~ 1 log reduction in lung bacterial burden compared to control animals treated with isotype control. These findings define a novel, structure-guided strategy to amplify the functional capacity of natural anti-*M. tb* antibodies and highlight bispecific antibody platforms as promising candidates for host-directed tuberculosis immunotherapy.

## Author summary

Tuberculosis (TB), caused by *Mycobacterium tuberculosis (M. tb)*, continues to be a major global health threat, claiming millions of lives every year. Despite being over a century old, the only available vaccine provides limited protection, and current treatment regimens are long, complex, and increasingly undermined by drug resistance. These challenges highlight the urgent need for innovative therapeutic strategies. One promising avenue is monoclonal antibody (mAb) therapy, which has already revolutionized the treatment of cancers and some viral infections. However, so far, antibodies generated during TB infection demonstrated only moderate protection, limiting their therapeutic potential. In this study, we harnessed antibody engineering to enhance the protective activity of human anti-TB antibodies. We focused on two antibodies, p4-36 and p4-163, that target a bacterial protein called PstS1. Using computational design, we improved the binding strength of p4-163, producing a more potent variant, p4-163LR. We further combined p4-36 and p4-163LR into a bispecific antibody capable of simultaneously recognizing two distinct sites on PstS1. This engineered antibody displayed superior ability to promote immune cell recognition of *M. tb* and significantly reduced bacterial burden in infected mice. Our findings provide proof-of-concept that antibody engineering can unlock new strategies for developing powerful immunotherapies against TB.

## Introduction

*Mycobacterium tuberculosis* (*M. tb*) remains a leading infectious killer, with tuberculosis (TB) affecting an estimated 10.8 million people and causing 1.23 million deaths in 2024 alone- nearly twice the mortality caused by HIV [1]. Transmission typically occurs through inhalation of aerosolized droplets, with *M. tb* primarily infecting alveolar macrophages in the lungs developing active pulmonary TB in approximately 5–10% of the cases [2,3], and can be more frequent in immunocompromised individuals [4].

T cell response is critical for *M. tb* control, as evidenced from T cell depletion experiments which increased reactivation and the appearance of more severe disease in *M. tb*-infected mice, macaques, and individuals with acquired immunodeficiencies [3,5]. The association between B cell depletion and increased bacterial burden is not straightforward, with studies reporting varying outcomes [6,7]. Recently, growing evidence highlights a more substantial role for B cells in *M. tb* infection than previously appreciated [8–10]. Antibodies purified from individuals who remain uninfected despite prolonged exposure to *M. tb* were shown to exhibit protection in mice from pathogenic *M. tb* challenge [11]. More recently, post-hoc analysis of the M72/AS01E subunit vaccine, which showed ~50% bacterial clearance, linked *M. tb* -specific antibody responses with protection [12].

Monoclonal antibodies (mAbs) isolated from infected individuals also demonstrated various degrees of efficacy in protecting from *M. tb* challenge. Anti-lipoarabinomannan antibodies correlate with protection from *M. tb* in both mice and in non-human primates [13,14]. LpqH-specific antibodies from exposed individuals substantially lower *M. tb* burden in a murine protection model [15]. Recently, a panel of mAbs was evaluated in mice and demonstrated approximately 20% to 50% reduction in bacterial loads [16]. Current evidence suggests that such antibodies can, in principle, restrict bacterial growth, by acting either directly on the bacterium itself or indirectly, by leveraging immune cells to reduce bacterial load. Nevertheless, the relatively modest protection conferred by all naturally elicited antibodies reported to date underscores their possible inherent limitations. Unlike the high potency often observed with antiviral mAbs, anti- *M. tb* antibodies have thus far demonstrated only limited CFU reduction, rarely exceeding 50%. This disparity suggests that developing strategies to enhance naturally elicited anti- *M. tb* mAbs through functional optimization and antibody engineering, may generate next-generation antibodies with markedly improved potency, durability, and even therapeutic potential.

Structure-guided computational approaches have been successfully employed to design antibodies with improved antigen affinity, specificity, and enhanced protein stability [17]. Recently, rational Fc-engineering empowered previously ineffective α-glucan–specific antibodies to mediate potent, neutrophil-dependent restriction of *M. tb* in whole-blood assays [9]. Similarly, rational engineering of the Fab part can be employed to reach higher antibody affinity, better avidity and enhanced potency [18], which has been employed in our study. We previously used single B cell sorting and cloning to isolate a panel of nine mAbs from a blood sample of a TB infected donor who was treated with antibiotics until recovery [19]. The individual, P004, exhibited strong serum reactivity against the *M. tb* phosphate transporter protein, PstS1. The most potent mAbs, members of two different clones, named p4-36 and p4-163, target two non-overlapping epitopes on the surface of PstS1 [19] and provide 30–40% protection when injected into mice prior to aerosol challenge with pathogenic *M. tb* [19]. The aim of the current study was to rationally improve the activity of these anti-PstS1 mAbs. For this purpose, we analyzed the atomic coordinates of a close clonal variant of the p4-163 mAb (p4-170) bound to PstS1, and employed a computational design approach to generate an improved antibody, named p4-163LR. We then generated antibodies that combine the specificities of both p4-163LR and p4-36, and generated a bispecific antibody (Bi-S 36/163LR), that when given prior to infection, reduces *M. tb* infection by approximately ~1 log10 in an aerosol challenge mouse model. Our results demonstrate that, beyond Fc region targeting, engineering the Fab region of anti-*M. tb* antibodies can significantly enhance their protective potency. Notably, we show that natural anti-*M. tb* antibodies can be modified to improve their functional efficacy, resulting in substantial reductions in bacterial burden.

## Results

### Computational prediction of improved p4-163 variants and isolation of p4-163LR

**Fig 1:**
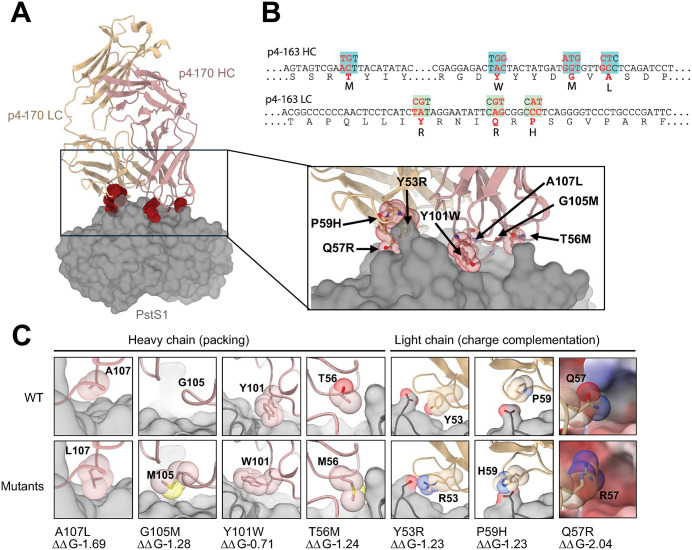
Generation of improved p4-163 mAbs variants. **(A)** Structure of the p4-163/170 Fab:PstS1 complex. Heavy chain (HC) is shown in pink, light chain (LC) is shown in tan, and PstS1 is shown in gray. Seven residue substitutions predicted to improve Fab binding to PstS1 are shown as red spheres. The insert shows a close-up view, the red spheres become semi-transparent, revealing the amino acid side chains depicted as sticks. The corresponding residues are labeled. **(B)** Nucleotide and amino acid sequences of the p4-163 heavy (HC) and light (LC) chains, where substitutions are highlighted in red. **(C)** Close-up comparisons between the original Fab:PstS1 complex (top) and the seven predicted variants (bottom). Predicted binding affinity improvements are attributed to better charge and surface complementation. FoldX predicted complex energy reduction (ΔΔG) for each variant is indicated at the bottom. The structure illustrations in **(A)-(C)** were created with publicly available PyMOL software.

The first stage in improving the binding of p4-163 to its cognate antigen PstS1 was to identify positions within the binding interface whose modification could increase mAb binding. The FoldX “BuildModel” algorithm [20] was applied to the high-resolution crystal structure of the p4-170 antibody (a close variant of p4-163) in complex with PstS1 (PDB: 7DM2, Fig 1A) [19] to systematically generate mutant constructs. Specifically, each amino acid residue within 5 Å of the antigen was individually substituted with the 19 alternative amino acids, resulting in an *in-silico* library of 570 (19 * 30) p4-163 variants. The modeling was repeated three times, and the corresponding changes in free energy (ΔG) were computed for each model. Based on these calculations, we identified four mutations in the heavy chain and three in the light chain that were predicted to improve binding affinity of the mAb to PstS1. Guided by these predictions, we engineered seven p4-163 mAb variants, each incorporating a single substitution in either the heavy or light chain, paired with the complementary wild-type chain (Fig 1B and 1C).

**Fig 2:**
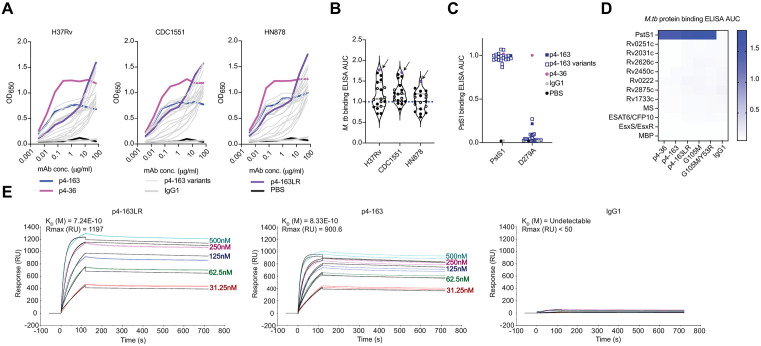
Binding of p4-163LR mAb. **(A)** Binding curves of generated p4-163 variants determined by ELISA to *M. tb* lysates H37Rv, CDC1551 and HN878 strains. p4-36 – magenta line, p4-163 – dark blue line, p4-163 variants – thin gray line, p4-163LR – purple line, IgG1 – thick gray line and PBS – black. **(B)** Area under the curve (AUC) as measured by ELISA for p4-163 variants bearing either a single substitution (IgH or IgL) indicated in solid black dots or double substitutions (IgH and IgL) indicated in empty black dots against the *M. tb* lysates H37Rv, CDC1551 and HN878 strains. p4-163LR is indicated in solid purple dots. The AUC of the parental p4-163 mAb (blue dashed line) was normalized to 1 and all the other variants were calculated accordingly. **(C)** AUC of the p4-163 variants as measured by ELISA for PstS1 and PstS1_D279A_. AUC has been normalized to the parent, p4-163. p4-36 – magenta dot, p4-163 – solid blue square, p4-163 variants – empty blue square, IgG1 – gray dot and PBS – black dot. **(D)** ELISA AUC heatmap depicting binding of p4-163 mAb variants against 11 recombinantly expressed *M. tb* proteins and maltose binding protein (MBP) from *E. coli* as control. Anti-PstS1 mAbs show specific binding only to PstS1. **(E)** Surface plasmon resonance (SPR) sensorgrams showing binding kinetics of mAbs to immobilized PstS1. Representative SPR response curves (response units, RU) are shown for mAbs, p4-163, p4-163LR and isotype control, IgG1 at increasing mAb concentrations (31.25, 62.5, 125, 250, and 500 nM) injected over the ligand-coated surface. The association phase is observed during mAb injection, followed by dissociation after return to running buffer. Colored curves represent experimental data and black lines indicate the global fit to a 1:1 Langmuir binding model.

ELISA assays indicated that six out of these seven p4-163 variants indeed bound *M. tb* lysates derived from strains H37Rv and CDC1551 better than the parental p4-163 antibody (S1 Fig). To test whether further improvements in binding could be identified, we generated 12 additional p4-163 variant mAbs by pairing each of the four heavy chain variants (each containing a single point mutation) with each of the three light chain variants (also containing a single point mutation). These double-mutant variants displayed a pronounced, dose-dependent binding to *M. tb* lysates than the original p4-163 antibody (Fig 2A). Among them, a variant featuring a Y53R substitution in the light chain and an A107L substitution (p4-163LR) in the heavy chain showed particularly improved binding (Fig 2A and 2B). Importantly, all p4-163 variants maintained sensitivity to the PstS1_D279A_ mutation, which abolishes binding to this antibody family [19], confirming that the antigen specificity remained unaltered (Fig 2C). To further exclude non-specific binding of the improved antibodies to non-PstS1 *M. tb* antigens, ELISA was performed against a panel of 10 recombinantly expressed *M. tb* proteins (Fig 2D). In parallel, surface plasmon resonance (SPR) analysis demonstrated that the engineered variant p4-163LR exhibits enhanced binding affinity compared to the parental antibody (Fig 2E). The enhanced binding affinity of p4-163LR is likely due to two structural changes: (1) the introduction of a new hydrophobic contact between L107_HC_ and PstS1, and (2) the formation of a salt bridge between R53_LC_ and E182_PstS1_, replacing the original van der Waals interaction between Y53_LC_ and P181_PstS1_ (S2 Fig). Together, these results demonstrate that structure-guided *in silico* design followed by Fab engineering can yield p4-163 variants with enhanced affinity for the *M. tb* antigen PstS1.

### The p4-163LR variant exhibits improved functions *in vitro* but not *in vivo*

**Fig 3:**
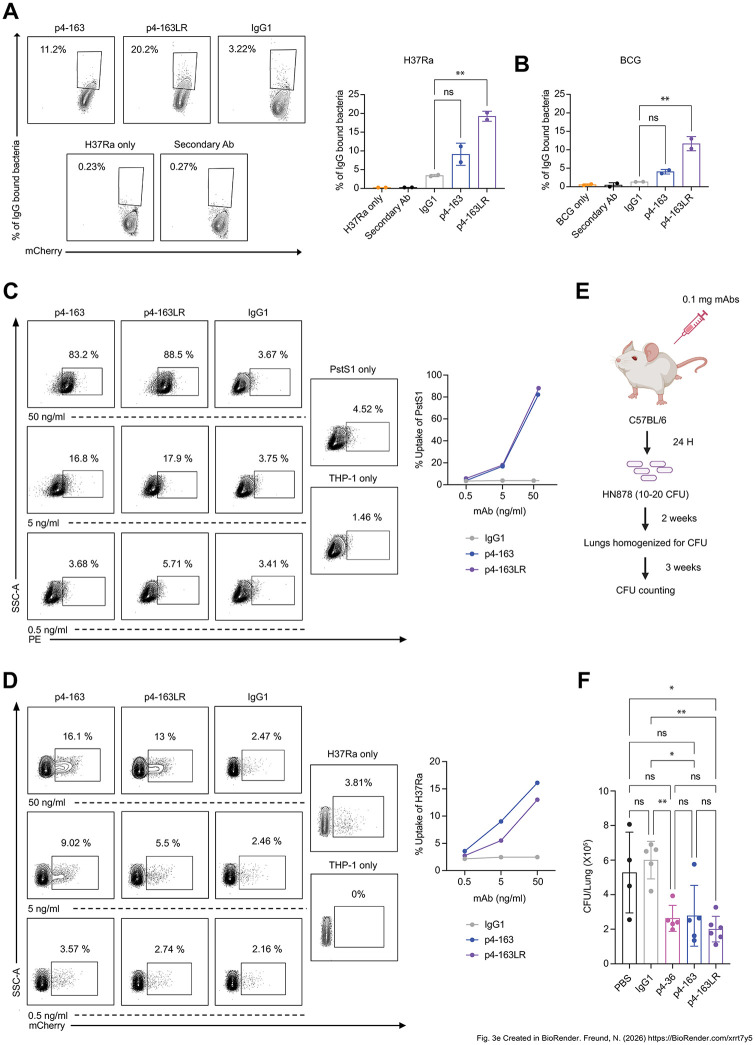
Binding and *in vivo* activity of p4-163LR mAb. **(A)** Binding of antibodies to live H37Ra-mCherry as determined by flow cytometry and quantification of binding percentage. Gating strategy is provided in S6A Fig. IgG1 serves as an isotype control. Error bars are represented as mean ±SD. Significance was determined by GraphPad Prism software using one-way ANOVA. ns: not significant, *: p-value < 0.05, **: p-value < 0.01, ***: p-value <0.001 and ****: p-value < 0.0001. **(B)** Quantification of binding of antibodies to live BCG-mCherry as determined by flow cytometry. IgG1 serves as an isotype control. Gating strategy and the flow plots are provided in S6C Fig. Error bars are represented as mean ± SD. Significance was determined by GraphPad Prism software using one-way ANOVA. ns: not significant, *: p-value < 0.05, **: p-value < 0.01, ***: p-value <0.001 and ****: p-value < 0.0001. Dose dependent antibody-mediated uptake of **(C)** PstS1-PE and **(D)** H37Ra-mCherry by THP-1 monocytic cells in the presence of antibodies: IgG1 isotype control, p4-163, and p4-163LR, by THP-1 monocyte cell line and as determined by flow cytometry. Gating strategy is provided in S6D and S6E Fig respectively. **(E)** Schematic of *M. tb* infection assay in C57BL/6 mice in the presence and absence of antibodies (Created in BioRender. Freund, N. (2026) https://BioRender.com/xrrt7y5). Mice were injected once, intra-peritoneally with antibodies (0.1 mg/mL) 24 h prior to aerosol infection with pathogenic *M. tb* strain HN878. **(F)** Lung *M. tb* bacterial burden determined as CFU at 2 weeks following *M. tb* infection. IgG1 control – gray, p4-36 – magenta, p4-163 – dark blue, p4-163LR – purple and PBS – black. Error bars are represented as mean ± SD. In each treatment n = 4-5 mice. Significance was determined by GraphPad Prism software by one-way ANOVA compared to IgG1 control antibody. ns: not significant, *: p-value < 0.05, **: p-value < 0.01, ***: p-value <0.001 and ****: p-value < 0.0001.

p4-163LR variant exhibits a 1.8-fold increase in binding to intact H37Ra-mCherry [19] – a fluorescent attenuated *M. tb* strain –compared with the parental mAb p4-163 (Fig 3A). Similarly, p4-163LR exhibited a 3-fold increase in binding to intact Bacillus Calmette-Guérin (BCG)-mCherry relative to the parental antibody (Fig 3B). The ability of anti*-M. tb* mAbs to mediate effector functions is thought to be important for their activity [21]. In our previous study we showed that anti-PstS1 antibodies promote uptake by THP-1 cells [19]. Consistent with these findings, both p4-163 and p4-163LR mediated dose-dependent uptake of PstS1 by THP-1 cells, reaching nearly 100% PstS1 at 50 ng/ml antibody (Fig 3C). Similarly, both p4 antibodies also promoted dose-dependent uptake of H37Ra, although with lower efficiency compared to uptake of the purified antigen (Fig 3D).

Once the phagocytic effect on the antibodies was confirmed, we sought to evaluate their efficacy at the whole organism level. Although no single animal model fully recapitulates human tuberculosis infection, established murine models remain essential tools for preclinical evaluation. Human IgG can engage murine Fc receptors and elicit effector functions in mice [22], supporting their use for *in vivo* evaluation. Accordingly, pre-exposure prophylaxis studies in the acute infection mouse model are often used to assess *in vivo* activity and to provide an initial indication of antibody efficacy against *M. tb* infection [19,23]. When tested for their ability to inhibit *M. tb*, in a prophylactic model using *M. tb*-infected C57BL/6 mice, p4-163LR showed a comparable ability to reduce lung bacterial burden, consistent with prior findings where p4-163 alone achieved a ~ 50% reduction (Fig 3E and 3F). These results demonstrate that the LR modification preserved the protective capacity of the antibody but did not confer additional benefit, highlighting that enhanced antigen binding alone does not necessarily translate into improved *in vivo* efficacy. This finding motivated us to explore further engineering approaches beyond affinity enhancement.

### Bi-S 36/163LR exhibits enhanced effector functions compared to their monospecific counterparts

**Fig 4:**
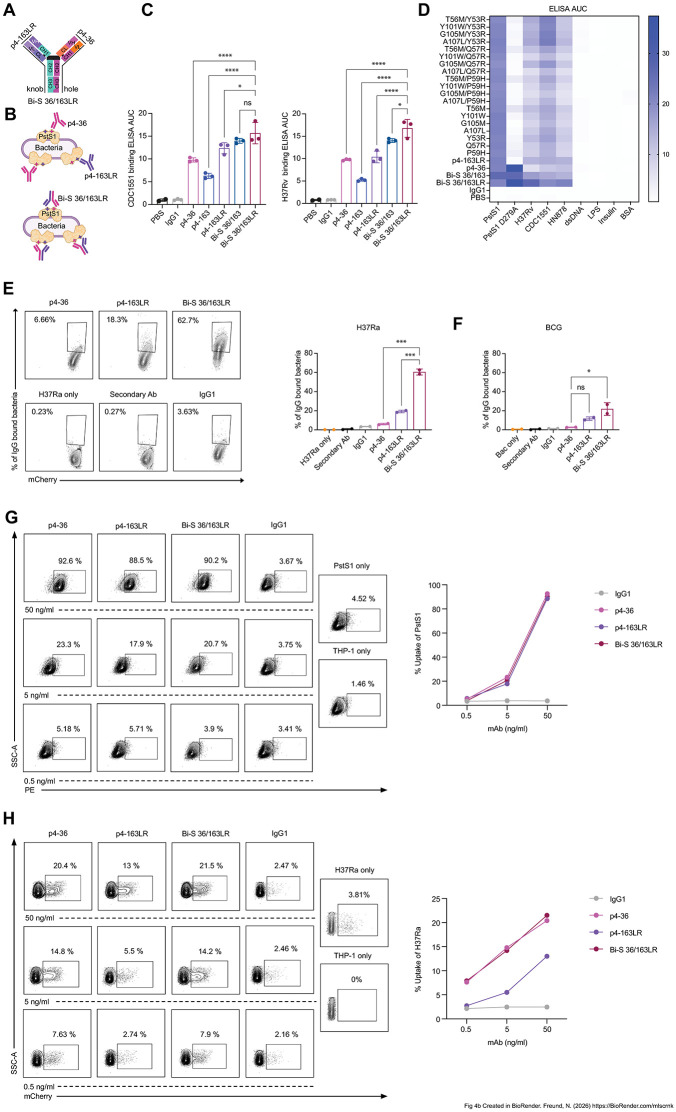
Engineering and binding of bispecific mAbs. **(A)** Engineering strategy for generating bispecific mAbs. The “knob in a hole” approach and the cross-mAb technology was utilized to generate Bi-S 36/163LR which harbors one arm of p4-36 and the second arm of p4-163LR. (B) A schematic illustrating how the bispecific antibody (Bi-S 36/163LR) targets simultaneously two epitopes on PstS1, enabling the formation of larger immune complexes that may enhance bacterial surface binding and ADCP compared with the parental antibodies, p4-36 and p4-163LR (Created in BioRender. Freund, N. (2026) https://BioRender.com/mlscrnk). **(C)** AUC as measured by ELISA for Bi-S 36/163 (carbon blue) and Bi-S 36/163LR (wine) along with their parental antibodies, p4-36 (magenta), p4-163 (dark blue) and p4-163LR (purple) against the *M. tb* lysates CDC1551 and H37Rv. Error bars are represented as mean ± SD. Significance was determined by GraphPad Prism software using one-way ANOVA. ns: not significant, *: p-value < 0.05, **: p-value < 0.01, ***: p-value <0.001 and ****: p-value < 0.0001. **(D)** ELISA AUC heatmap depicting specific binding of the anti-PstS1 mAbs. Note that our engineered mAbs do not exhibit polyreactivity as confirmed using controls such as dsDNA, LPS, insulin and BSA. **(E)** Binding of antibodies to live H37Ra-mCherry as determined by flow cytometry. Gating strategy same as provided in S6A Fig. IgG1 serves as an isotype control. Error bars are represented as mean ± SD. Significance was determined by GraphPad Prism software using one-way ANOVA. ns: not significant, *: p-value < 0.05, **: p-value < 0.01, ***: p-value <0.001 and ****: p-value < 0.0001. **(F)** Quantification of binding of antibodies to live BCG-mCherry as determined by flow cytometry. IgG1 serves as an isotype control. Gating strategy and the flow plots are provided in S6C Fig. Error bars are represented as mean ± SD. Significance was determined by GraphPad Prism software using one-way ANOVA. ns: not significant, *: p-value < 0.05, **: p-value < 0.01, ***: p-value <0.001 and ****: p-value < 0.0001. Dose dependent antibody-mediated uptake of **(G)** PstS1-PE and **(H)** H37Ra-mCherry as determined by flow cytometry of THP-1 monocyte cells in the presence of antibodies: IgG1 isotype control, p4-36, p4-163LR and Bi-S 36/163LR by THP-1 monocyte cell line and as determined by flow cytometry. Gating strategy same as provided in S6D and S6E Fig respectively.

Anti-PstS1 mAbs p4-36 and p4-163 target distinct, non-overlapping epitopes of PstS1 [19] (S3 Fig). We therefore next examined whether a bispecific antibody that concomitantly targets both binding sites would exhibit improved functional activity. For this purpose, we applied the “knob into hole” and cross-mAb approach [24] to generate a bispecific antibody, Bi-S 36/163LR, which harbors one arm of p4-36 mAb and the second arm of p4-163LR mAb (Fig 4A and 4B). As anticipated, the bispecific antibodies effectively bound to both PstS1_D279A_ and PstS1_K136E_ mutant forms of PstS1- mutations that individually abrogate binding of p4-163 and p4-36, respectively, while retaining binding to the PstS1, thereby confirming their bispecific nature (S4 Fig). Bi-S 36/163LR exhibited enhanced binding to pathogenic *M. tb* strains, H37Rv, CDC1551 and HN878 with Bi-S 36/163LR binding slightly better than Bi-S 36/163 (harboring one arm of p4-36 mAb and the second arm of p4-163 mAb) to H37Rv (Fig 4C and 4D). This improvement was not due to increased polyreactivity, as measured by ELISA, against dsDNA, LPS, insulin and BSA (Fig 4D). Notably, Bi-S 36/163LR demonstrated a ~ 3-fold and ~10-fold increase compared to p4-163LR and p4-36, respectively in binding to intact H37Ra-mCherry (Fig 4E). Consistently, Bi-S 36/163LR showed a modest but reproducible increase in binding to intact BCG-mCherry relative to both monospecific antibodies (Fig 4F). While Bi-S 36/163LR did not enhance uptake of purified PstS1 or H37Ra compared with p4-36, it mediated moderately improved bacterial uptake relative to p4-163LR (Fig 4G and 4H).

### Bi-S 36/163LR is protective in mice

**Fig 5:**
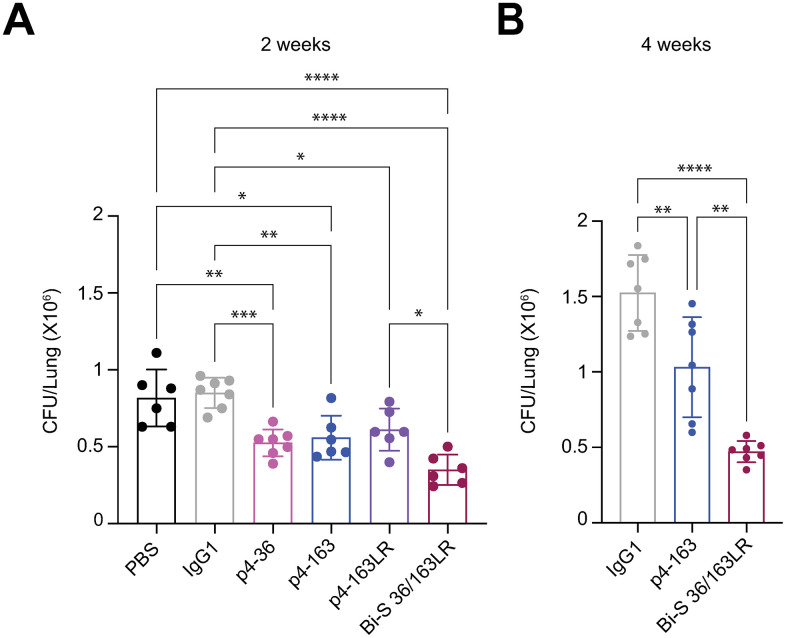
*In vivo* activity of bispecific mAbs. *M. tb* bacterial burden in C57BL/6 mice in the presence and absence of antibodies. Mice were injected once, intra-peritoneally with antibodies (0.1 mg/mL) 24 h prior to aerosol infection with pathogenic *M. tb* strain HN878. Lung *M. tb* bacterial burden was determined as CFU at **(A)** 2 weeks and **(B)** 4 weeks following *M. tb* infection. IgG1 control – gray, p4-36 – magenta, p4-163 – dark blue, p4-163LR – purple, Bi-S 36/163LR – wine and PBS – black. Error bars are represented as mean ± SD. In each treatment n = 4-5 mice. Significance was determined by GraphPad Prism software by one-way ANOVA. ns: not significant, *: p-value < 0.05, **: p-value < 0.01, ***: p-value <0.001 and ****: p-value < 0.0001.

To assess whether Bi-S 36/163LR has an enhanced protective capacity, we evaluated its effect on bacterial burden in the prophylactic *in vivo* model as described before. C57BL/6 mice were injected with the mAbs p4-36, p4-163 and p4-163LR, the Bi-S 36/163LR or isotype control prior to infection with pathogenic *M. tb,* and lung bacterial burdens were quantified at 14 days post-infection. As before, p4-36 and p4-163 reduced bacterial burdens by ~40% compared with isotype control, and p4-163LR had similar levels of protection (Fig 5A). In this assay, Bi-S 36/163LR conferred significantly enhanced protection compared with the monospecific PstS1 mAbs by reducing bacterial burdens by an additional 20% (Figs 5A and S5), suggesting that Bi-S 36/163LR contributes to improved bacterial clearance *in vivo*. This effect was maintained even when testing at later time point of 4 weeks post infection (Fig 5B). Remarkably, mice injected with Bi-S 36/163LR had a full 1 log10 reduction in lung bacterial burden compared with isotype control. These results underscore the superior functional activity of Bi-S 36/163LR, demonstrating its improved efficacy *in vivo*. These findings suggest that Bi-S 36/163LR achieves enhanced functional engagement with the antigen owing to its ability to target multiple epitopes of PstS1. This dual specificity may facilitate the recruitment of coordinated effector mechanisms, opening exciting avenues for further exploration.

## Discussion

Unlike the potent neutralizing mAbs reported to be isolated from individuals infected with viruses, such as HIV [25–27], SARS-CoV-2 [21,28,29], or Mpox [30,31], most reported anti-*M. tb* antibodies demonstrate only modest efficacy [32] suggesting that their activity could be further optimized. The elicited antibody responses can be perhaps improved through targeted immunization strategies that strategically expose the immune response towards favorable antigenic sites. In parallel, molecular engineering offers an avenue to augment the functional capacity of naturally elicited antibodies, leveraging their inherent potential as a starting point to optimize efficacy and unlock stronger, more durable effects. Prior efforts have focused on optimizing Fc-mediated effector functions [9], where an otherwise non-protective antibody targeting *M. tb* α-glucan was converted into a functional antimicrobial agent via Fc engineering. Our strategy adopts a distinct and complementary approach by targeting the antigen-binding (Fab) region. We therefore demonstrate, for the first time, that structure-guided Fab design principles can be successfully applied to antibodies targeting *M. tb*. These findings establish Fab-focused engineering as a viable and rational strategy to enhance *M. tb* antibody performance and to achieve the level of potency likely required for clinical efficacy.

In our study, we specifically employed structure-guided *in silico* modeling to introduce rational mutations into the paratope of a naturally occurring antibody, thereby enhancing its affinity to the antigen and, subsequently, its functional performance. This strategy draws conceptual inspiration from the field of antiviral antibody optimization, where mAbs have been optimized to achieve 10–50-fold improvement in potency [33,34]. However, in our case, *in silico* modeling involving substitution of two residues within the antigen-binding region did not result in a substantial enhancement of protective efficacy. Consequently, we introduced a second, key modification, which is engineering of a bispecific antibody targeting a second, non-overlapping epitope on the same antigen. Together, these enhancements led to a 2–3-fold improvement in protective efficacy *in vivo* compared to the parental monospecific p4-163. Although modest, this increase in potency is biologically meaningful and likely reflects the ability of the bispecific antibody to engage the target through both binding arms, thereby compensating for the relatively low surface abundance of PstS1 and the competitive presence of numerous other antigens on *M. tb*. It was reported that the bispecific antibody configuration enables the antibody to more efficiently mediate effector functions and enhance immune activation through improved recruitment of effector cells, such as NK cells [35], as well as promote a more efficient clearance by macrophages [36]. Antibody-mediated reduction of bacterial dissemination through Fc-mediated activation of the innate immune response can lead to complement-induced bacteriolysis, which has been demonstrated for other antimicrobial antibodies [37–39]. Lastly, in humans, antibodies were demonstrated to reshape the immune response resulting in activating CD8 T cells contributing to post antiretroviral interruption viral control of HIV-1 [40].

A limitation of the present study is the lack of detailed immune correlates and antibody infiltration measurements to infected lungs associated with protection in antibody-treated mice. While a ~ 1-log reduction in bacterial burden following antibody treatment demonstrates substantial biological activity, CFU reduction alone is not always a definitive predictor of improved pathology. Antibodies can confer survival or pathological benefits independent of changes in bacterial load, suggesting antibody mediated changes in the host immune responses or disease pathology in ways not captured by CFU measurements alone [41]. Future studies will address these gaps by systematically evaluating immune signatures, inflammation, and histopathological changes to better define the mechanisms underlying antibody-mediated protection.

Lastly, while the magnitude of bacterial reduction observed in our study is comparable to that reported for several host-directed therapeutic candidates [42,43], our antibodies were evaluated in a prophylactic rather than in a therapeutic setting, which would be clinically relevant. While further studies will be required to evaluate the therapeutic potential of these antibodies in established infection models, our findings demonstrate that rational engineering of naturally elicited antibodies can enhance their protective activity against *M. tb*. Together, these results provide a foundation for the future development of antibody-based strategies to combat tuberculosis.

## Methods

### Ethics statement

All animal experiments with *M. tb* were performed in accordance with the guidelines of the Institutional Animal Care and Use Committee (IACUC) at the University of California, San Francisco (UCSF), under approved protocol number AN199620. Mice were housed and all procedures conducted in UCSF ABSL-3 facilities under institutionally approved biosafety protocols. All procedures complied with NIH guidelines, and every effort was made to minimize animal suffering.

### Computational prediction

FoldX algorithm was used on the atomic structure of p4-170 Fab:PstS1 (PDB: 7DM2), that was further optimized by FoldX “repairPDB” [20]. In the interface or within 5 Å of PstS1, the amino acids were replaced (one at a time) using the FoldX “BuildModel”, by the other 19 amino acids to provide an in-silico library of p4-163/p4-170 variants. Validation of the predicted variants was by running a simulation in triplicates. For testing the interaction between PstS1 and the predicted variants, the difference in binding energy (ΔG) was calculated between the complexes of the original antibody:PstS1 and the predicted variant:PstS1 (ΔGoriginal complex - ΔGvariant complex >1). Variants with a significant reduction in ΔG were selected for antibody production.

### Generation of antibody variants

Point mutations were introduced by PCR in the IgH or IgL of p4-163. Purified PCR products (PCR purification kit, Life Technologies) were digested to remove the parent methylated DNA using *dpnI* restriction enzyme (NEB), followed by another purification step and transformation into *Escherichia coli* (*E. coli)* DH5αF^-^ competent cells (NEB). All variants were validated by sequencing. For expression of antibodies, Expi293F mammalian cells (ThermoFisher Scientific), exponentially growing in a shaking incubator at 8% CO_2_ and 37 °C, were transiently transfected using the ExpiFectamine 293 transfection kit (ThermoFisher Scientific) according to the manufacturer’s protocol. A ratio of 1:2 was applied for pairing the IgH and IgL DNA expression vectors, respectively. The Bi-S Abs were expressed by pairing four expression vectors: p4-36 ‘hole’ IgH and its corresponding p4-36 CrossMab Igλ along with p4-163 ‘knob’ IgH and its corresponding parent p4-163 Igλ or the p4-163HC_A107L_ ‘knob’ IgH with its corresponding p4-163LC_Y53R_ Igλ in a ratio of 2:2:1:1. Seven days after transfection, the filtered (0.22 mm) cell supernatant was incubated for 2 hours at room temperature with MabSelect protein A agarose beads (Cytiva). The antibodies were purified by affinity chromatography and eluted with 50 mM sodium phosphate (pH = 3), normalized with 1 M Tris-HCl (pH = 8), that followed by buffer exchange dialysis into PBS × 1. The antibodies were aliquoted and stored at -80 °C.

### Bacterial strains and lysates

Lysates of the *M. tb* pathogenic bacteria, strain H37Rv (Cat # NR-14822), strain CDC1551 (Cat # NR-14823) and strain HN878 (Cat # NR-14824) were obtained from BEI Resources https://www.beiresources.org/. The fluorescent *M. tb* strains, H37Ra-mCherry and BCG-mCherry were a kind gift by Prof. Daniel Barkan, The Hebrew University of Jerusalem, and grown in Middlebrook 7H9 broth (Difco) containing 10% OADC, 0.05% Tween-80, and 0.5% glycerol. The H37Ra bacterial culture was incubated at 37 °C, with shaking at 120 rpm.

### Expression of *M. tb* recombinant proteins

The publicly available DNA sequence of the *M. tb* protein, PstS1 (Rv0934) and the mutant PstS1_D279A_ were designed in pMALp expression vectors and produced in *E. coli* (strain BL21, NEB) as described previously [19]. Following purification using affinity chromatography, the proteins were eluted with a solution containing 200 mM imidazole (Sigma) and 8 M urea (Bio-Lab) in PBS × 1. The urea was gradually removed by buffer exchange dialysis into PBS × 1 and the proteins were aliquoted and stored at -80 °C.

### ELISA

ELISA high-binding 96-well plates (Corning) were coated overnight at 4 °C with 1 μg/ml PstS1 or the point mutants PstS1_K136E_ and PstS1_D279A_, in addition to 10 μg/ml lysates of *M. tb* pathogenic bacteria, H37Rv, CDC1551 and HN878 strains (BEI Resources). The following day, the plates were washed three times with PBS × 1 containing 0.05% Tween-20 (Sigma) and blocked with “blocking buffer” consisting of 3% bovine serum albumin (BSA, MP Biomedicals), 20 mM EDTA, and 0.05% Tween-20 in PBS × 1, for 2 h at room temperature. Blocking was followed with 1 h incubation at room temperature with the indicated antibodies, starting at a concentration of 1-0.06 μg/ml (PstS1 coating) or 64–10 μg/ml (*M. tb* lysates coating) in consecutive 4-fold dilutions in PBS × 1. Followed by an additional washing step, the plates were incubated at room temperature in “blocking buffer” containing 0.16 μg/ml horseradish peroxidase (HRP)-conjugated goat anti-human IgG (Jackson ImmunoResearch, 109-035-088). The plates were developed after another washing step by the addition of 100 μl/well TMB/E substrate (Abcam) for 10 minutes at room temperature. The plates were read in an ELISA plate reader (BioTek 800 TS) at OD_650_.

### Biacore (Surface Plasmon Resonance, SPR)

All biacore experiments were performed at 25 °C using the Biacore T200 instrument. 5 μg/ml Capture Select Biotin Anti-IgG-Fc (Multi-species) conjugate (ThermoFisher Scientific) was immobilized for 600 s and a flow rate of 10 μl/min on sensor chip SA, series S (GE Healthcare). A sample of 0.25 μg/ml antibodies was injected over the chip bound Capture Select for 600 s and a flow rate of 10 μl/min. This was followed by an injection of PstS1 at five concentrations: 31.25, 62.5, 125, 250 and 500 nM. After each cycle, the chip was regenerated by the passage of 0.1 M glycine, pH = 2 at a flow rate of 30 μl/min for 1.5 min. Each antibody was tested in three replicates. The samples were diluted in HBS-EP buffer (0.01 M HEPES, 0.15 M NaCl, 0.003 M EDTA, and 0.05% Tween-20, pH = 7.4). Sensorgrams were generated for each sample and fitted to a 1:1 binding model by using nonlinear regression in the BIA evaluation software. K_D_ was measured by the ratio of dissociation (K_d_) and association (K_a_) kinetic constants, K_D_ = K_d_/K_a_.

### Bacterial staining

For assessment of direct binding of antibodies to the live *M. tb* strain, H37Ra-mCherry or BCG-mCherry, bacteria were grown to an optimal density of OD_600_ = 2. 1-2x10^8^ colony-forming units (CFUs) of bacteria were centrifuged for each sample, washed with PBS × 1, and incubated at room temperature for 1 h with or without 50 μg/ml antibodies as indicated. Next, the mixture of bacteria-antibody was centrifuged, washed with FACS buffer containing 1% fetal bovine serum (FBS) and 2 mM EDTA in PBS × 1, and fixed according to the manufacturer’s protocol (Tonbo Bioscience). The bacteria bound antibodies were then incubated with anti-human IgG-FITC (1:50 dilution, Miltenyi Biotec) for 20 min on ice and the unbound antibodies were removed by washing. All samples were resuspended in FACS buffer, passed through a CytoFLEX S4 flow cytometer (Beckman Coulter), and analyzed using FlowJo software.

### Antibody Dependent Cellular Phagocytosis (ADCP)

For assessment of ADCP by monocytes, the human monocyte THP-1 cell line (ATCC TIB-202) was cultured in RPMI-1640 medium (Biological Industries) supplemented with 10% heat-inactivated FBS, 1% penicillin-streptomycin, and 1% L-glutamine. The live *M. tb* strain, H37Ra-mCherry was grown as previously described and a total of 3x10^7^ bacterial cells were incubated with or without antibodies using three different concentrations, 50 ng/ml, 5 ng/ml and 0.5 ng/ml for 1 h at room temperature. Similarly, fluorescently labelled antigen, PstS1-PE was incubated with or without antibodies using the same antibody concentrations as indicated above. The bacteria-antibody mix or antigen-antibody mix was then added to pre-washed (RPMI-1640) 1x10^6^ non-activated THP-1 monocytes (MOI 30), and incubated for 3 h at 37 °C, 5% CO_2_. The cells were then washed twice with RPMI-1640, and incubated for 1 h at 37 °C, 5% CO_2_ with 250 μg/ml Amikacin sulfate salt (Sigma). Following an additional washing step, the cells were fixed according to the manufacturer’s protocol. All samples were passed through a CytoFLEX S4 flow cytometer and analyzed using FlowJo software.

### Mice *M. tb* -infection assay

Seven- to nine-week-old male and female C57BL/6 mice were used to evaluate the prophylactic efficacy of anti-PstS1 antibodies. Mice were injected intraperitoneally with 0.1 mg of antibody or isotype control 24 hours prior to infection. The following day, animals were exposed to a low-dose aerosol challenge with *M. tb* strain HN878 (10–20 CFU) using an inhalation exposure system. At either day 14 or day 28 post-infection, mice were euthanized and their lungs were aseptically harvested and homogenized. Serial dilutions of lung homogenates were plated on 7H11 agar plates supplemented with OADC and the PANTA antibiotic mixture. Plates were incubated at 37 °C, and CFUs were enumerated after 21 days.

## Supporting information

S1 Fig Binding of p4-163 variants to *M. tb* lysates. Binding curves of antibodies: p4-163 variants (thin gray line), p4-163 (bold blue line), p4-36 (bold magenta line) and IgG1 (bold gray line) against the *M. tb* lysates H37Rv and CDC1551 as measured by ELISA. PBS (bold black line) represents the negative control.
(TIF)

S2 Fig Structural and functional improvements in the p4-163LR variant. Structure showing close-up comparisons between the heavy and light chain mutations in p4-163LR. The left panel shows the formation of a salt bridge between R53_LC_ and E182_PstS1_, replacing the original van der Waals interaction between Y53_LC_ and P181_PstS1_ and the right panel shows the introduction of a new hydrophobic contact between L107_HC_ and PstS1.
(TIF)

S3 Fig Distinct epitopes recognized by the two monoclonal antibodies on PstS1. Structural mapping of the two monoclonal antibodies, p4-36 (magenta, PDB: 7DM1) and p4-170 (a clonal relative of p4-163, dark blue, PDB: 7DM2) onto the surface of the PstS1 protein (gray) illustrating their epitope locations.
(TIF)

S4 Fig Binding of mAbs to PstS1 mutants. Binding curves of anti-Pst1 antibodies: p4-36 (magenta), p4-163 (dark blue), p4-163LR (purple), Bi-S 36/163 (carbon blue), Bi-S 36/163LR (wine) and IgG1 (gray) against WT PstS1 and its mutants, PstS1_K136E_ and PstS1_D279A_ as measured by ELISA. The mutation K136E abrogates binding of p4-36 to PstS1 but not for others while the mutation D279A abrogates binding of p4-163 to PstS1. Bi-specific mAbs harboring one arm of p4-36 and other arm of p4-163/163LR retain binding to both the mutants.
(TIF)

S5 Fig Bi-S 36/163LR exhibits improved protection *in vivo* 2 weeks after infection. (A) *M. tb* bacterial burden in C57BL/6 mice in the presence of p4-36, p4-163LR and Bi-S 36/163LR. Mice were injected once, intra-peritoneally with antibodies (0.1 mg/mL) 24 h prior to aerosol infection with pathogenic *M. tb* strain HN878. Lung *M. tb* bacterial burden was determined as CFU at 2 weeks following *M. tb* infection. Error bars are represented as mean ± SD. In each treatment n = 6–7 mice. Significance was determined by GraphPad Prism software using one-way ANOVA. p = 0.002 between p4-163LR and Bi-S 36/163LR and p = 0.0265 between p4-36 and Bi-S 36/163LR. (B) *M. tb* bacterial burden in C57BL/6 mice in the presence of Bi-S 36/163 and Bi-S 36/163LR. Mice were injected once, intra-peritoneally with antibodies (0.1 mg/mL) 24 h prior to aerosol infection with pathogenic *M. tb* strain HN878. Lung *M. tb* bacterial burden was determined as CFU at 2 weeks following *M. tb* infection. Error bars are represented as mean ± SD. In each treatment n = 6 mice. Significance was determined by GraphPad Prism software using Welch’s t-test analysis (p = 0.0215).
(TIF)

S6 Fig Flow plots for bacterial staining and ADCP experiments. (A) Gating strategy for binding of anti-PstS1 mAbs to live H37Ra-mCherry as determined by flow cytometry. (B) Binding of mAbs to live whole bacteria, H37Ra using two different mAb concentrations, 50 μg/ml and 25μg/ml for p4-36, p4-163LR and Bi-S 36/163LR along with the isotype control. Top panel represents the gating strategy while the lower panels represent the % of IgG-bound bacteria. (C) Gating strategy (top) and flow plots (bottom) for binding of anti-PstS1 mAbs to live BCG-mCherry as determined by flow cytometry. Gating strategy for Antibody-mediated uptake of (D) PstS1-PE and (E) H37Ra-mCherry as determined by flow cytometry of THP-1 monocytic cells.
(TIF)

S1 Data An Excel file containing the underlying data for all figures presented in the manuscript. (XLSX)
