# Supplementary figures and images for: Modified Anti-PstS1 Bi-specific antibodies unlock potent protection against tuberculosis
Source: PLoS Pathog. 2026 May 27;22(5):e1014133. doi: 10.1371/journal.ppat.1014133 (PMC13215483; doi:10.1371/journal.ppat.1014133)

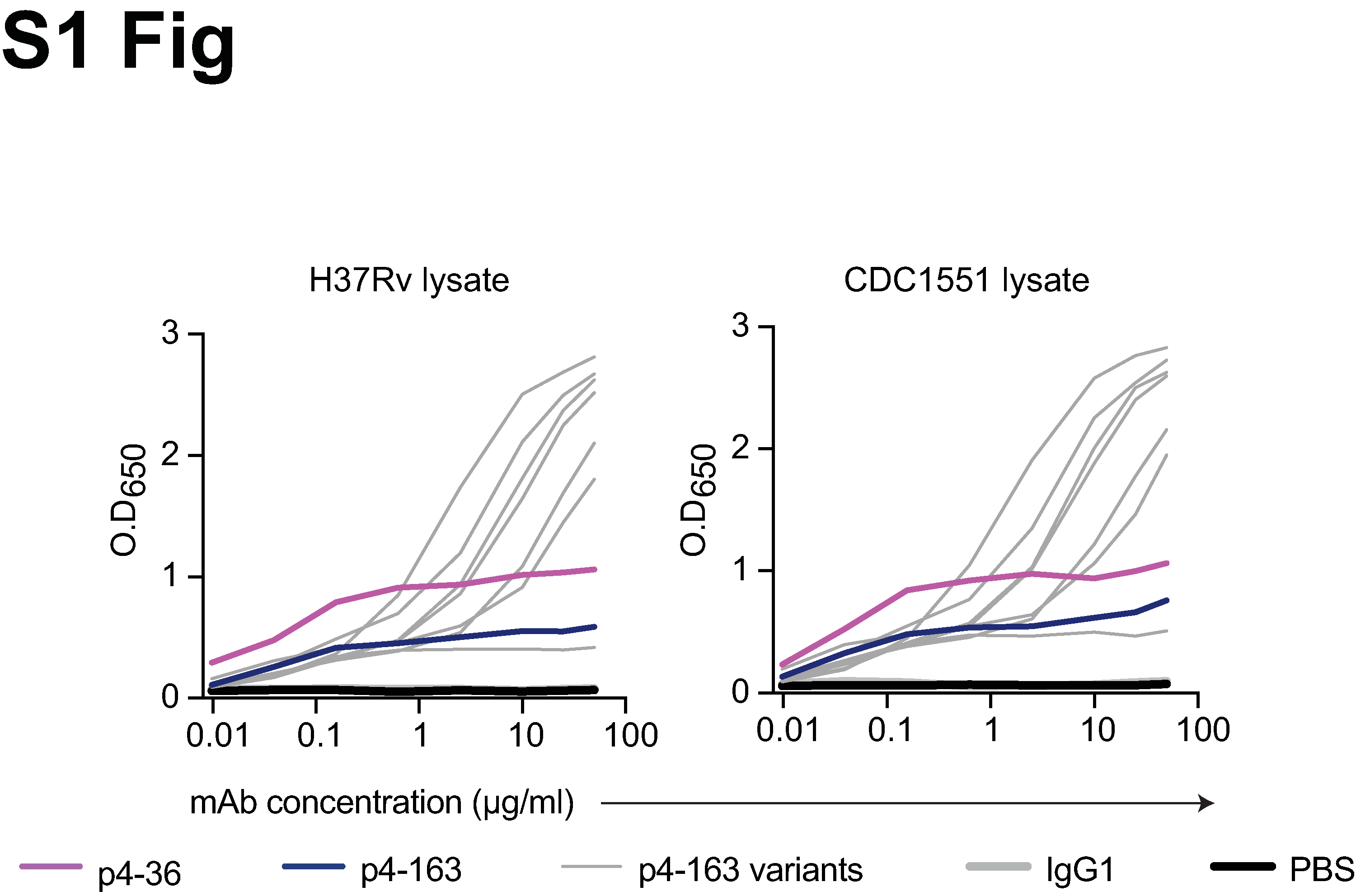

Supplement: S1 Fig — Binding curves of antibodies: p4-163 variants (thin gray line), p4-163 (bold blue line), p4-36 (bold magenta line) and IgG1 (bold gray line) against the M. tb lysates H37Rv and CDC1551 as measured by ELISA. PBS (bold black line) represents the negative control. (TIF) [file ppat.1014133.s001.tif]

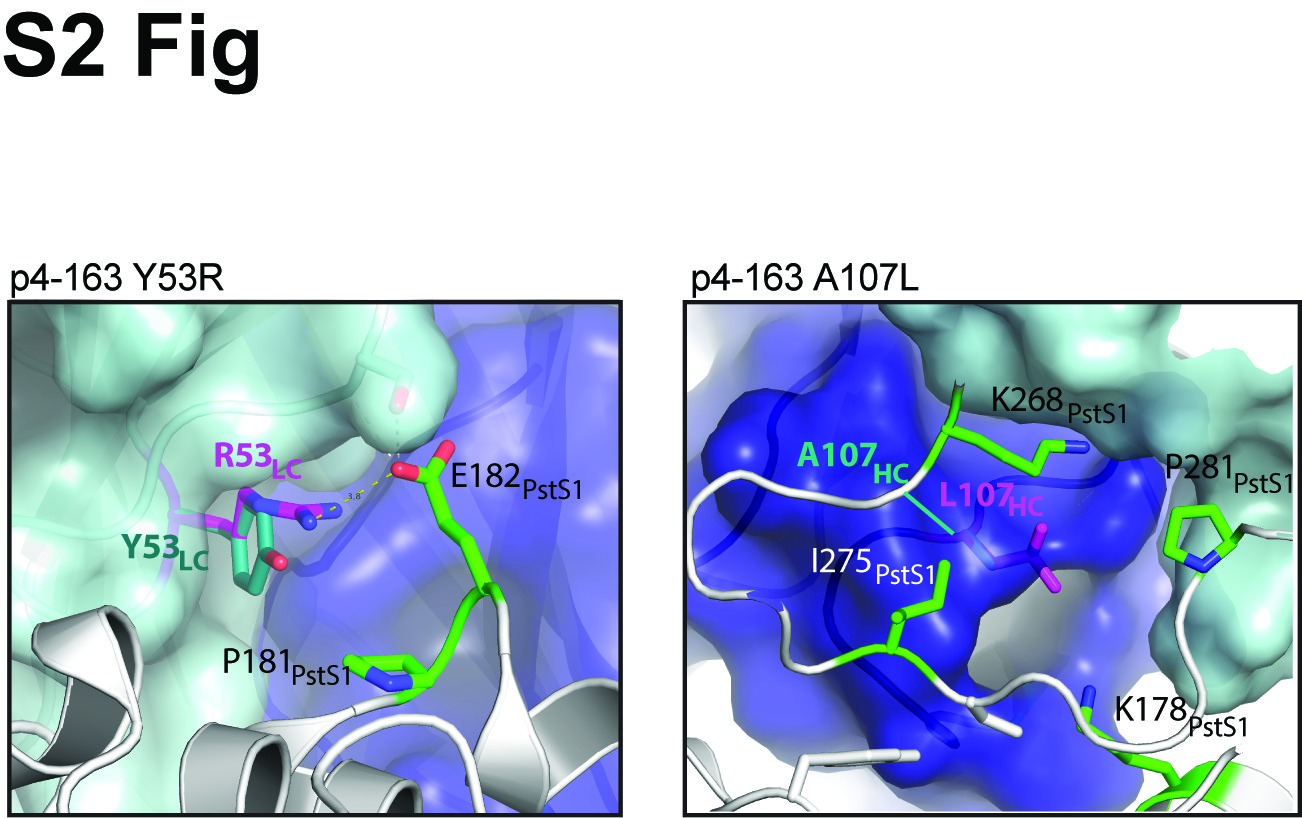

Supplement: S2 Fig — Structure showing close-up comparisons between the heavy and light chain mutations in p4-163LR. The left panel shows the formation of a salt bridge between R53LC and E182PstS1, replacing the original van der Waals interaction between Y53LC and P181PstS1 and the right panel shows the introduction of a new hydrophobic contact between L107HC and PstS1. (TIF) [file ppat.1014133.s002.tif]

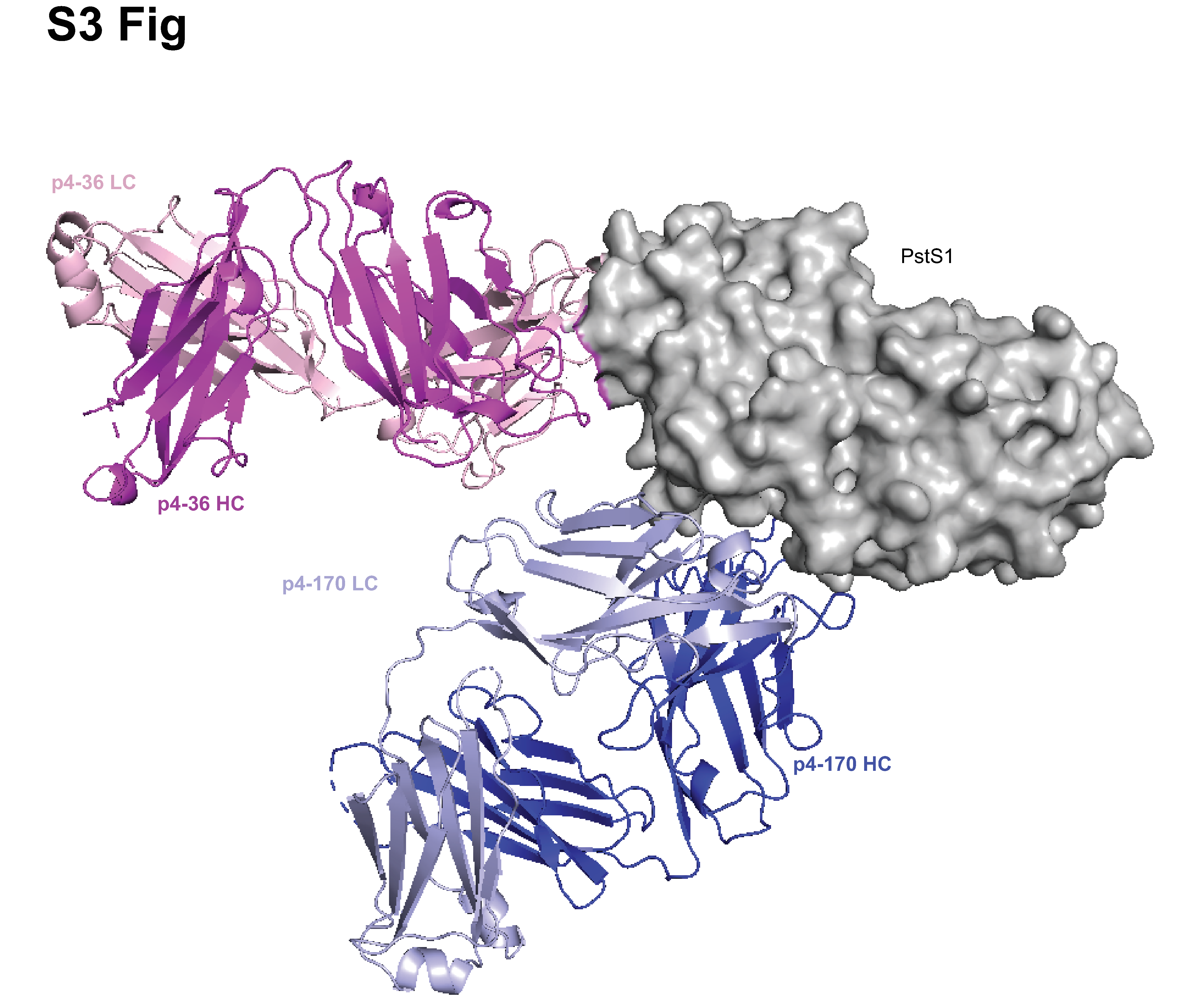

Supplement: S3 Fig — Structural mapping of the two monoclonal antibodies, p4-36 (magenta, PDB: 7DM1) and p4-170 (a clonal relative of p4-163, dark blue, PDB: 7DM2) onto the surface of the PstS1 protein (gray) illustrating their epitope locations. (TIF) [file ppat.1014133.s003.tif]

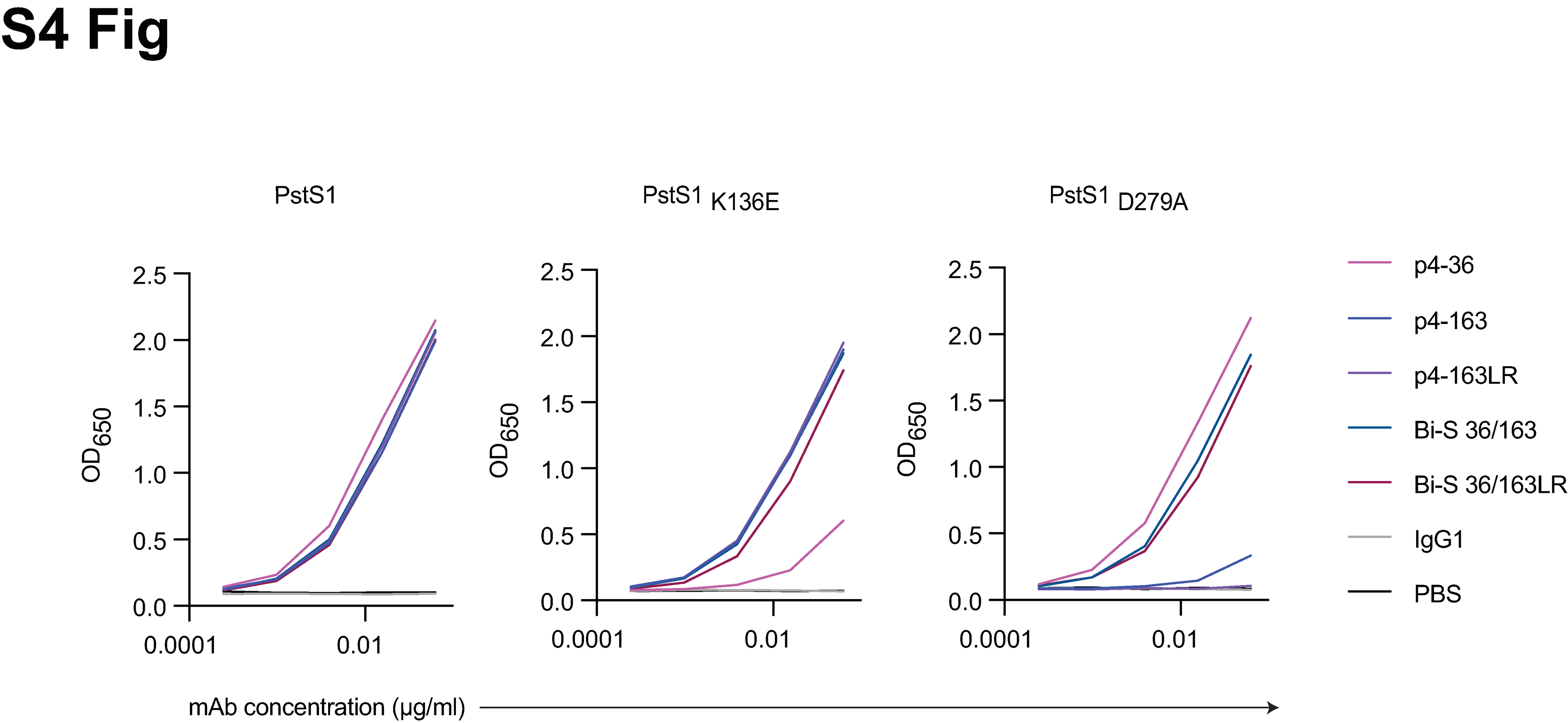

Supplement: S4 Fig — Binding curves of anti-Pst1 antibodies: p4-36 (magenta), p4-163 (dark blue), p4-163LR (purple), Bi-S 36/163 (carbon blue), Bi-S 36/163LR (wine) and IgG1 (gray) against WT PstS1 and its mutants, PstS1K136E and PstS1D279A as measured by ELISA. The mutation K136E abrogates binding of p4-36 to PstS1 but not for others while the mutation D279A abrogates binding of p4-163 to PstS1. Bi-specific mAbs harboring one arm of p4-36 and other arm of p4-163/163LR retain binding to both the mutants. (TIF) [file ppat.1014133.s004.tif]

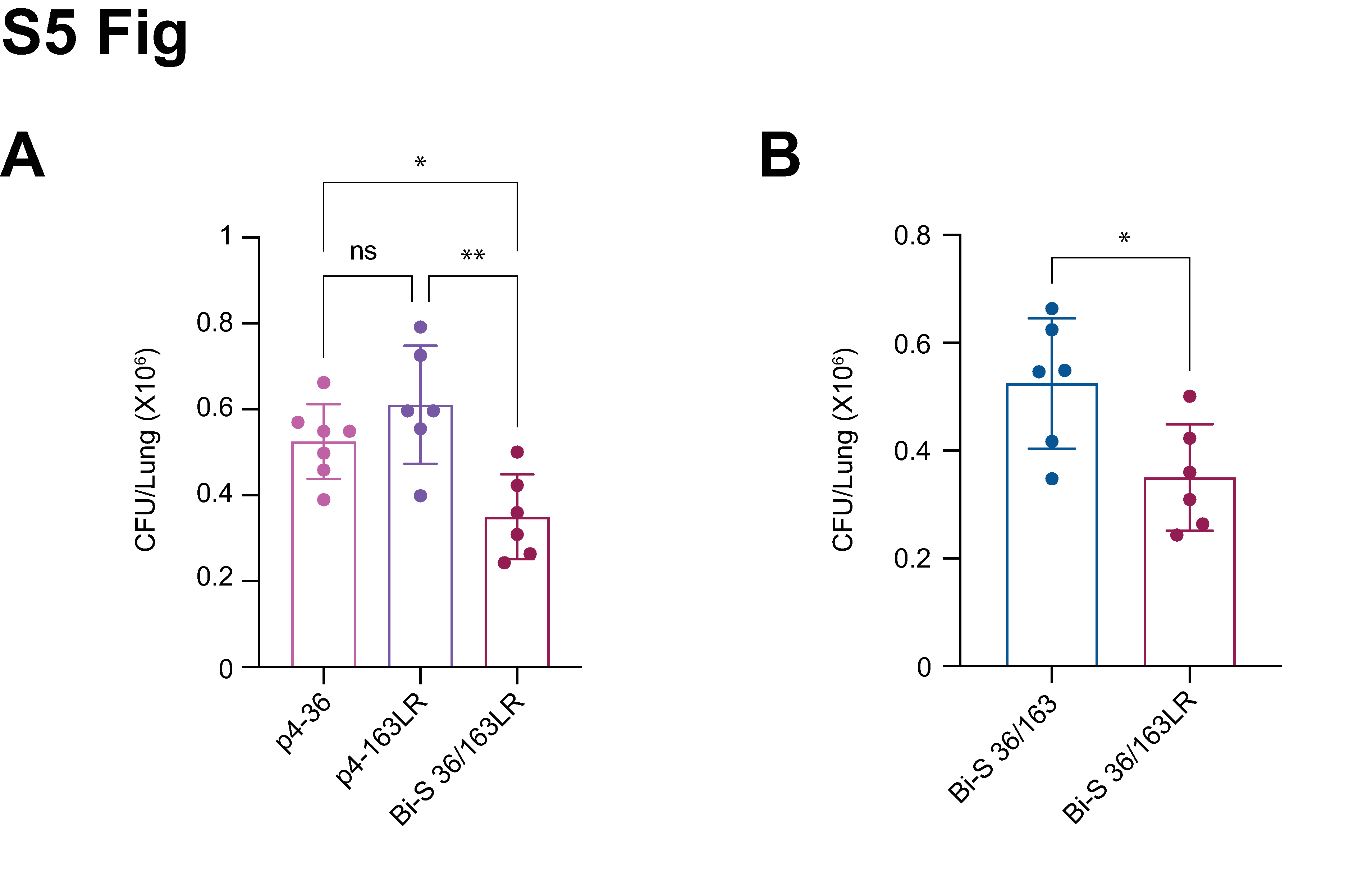

Supplement: S5 Fig — (A) M. tb bacterial burden in C57BL/6 mice in the presence of p4-36, p4-163LR and Bi-S 36/163LR. Mice were injected once, intra-peritoneally with antibodies (0.1 mg/mL) 24 h prior to aerosol infection with pathogenic M. tb strain HN878. Lung M. tb bacterial burden was determined as CFU at 2 weeks following M. tb infection. Error bars are represented as mean ± SD. In each treatment n = 6–7 mice. Significance was determined by GraphPad Prism software using one-way ANOVA. p = 0.002 between p4-163LR and Bi-S 36/163LR and p = 0.0265 between p4-36 and Bi-S 36/163LR. (B) M. tb bacterial burden in C57BL/6 mice in the presence of Bi-S 36/163 and Bi-S 36/163LR. Mice were injected once, intra-peritoneally with antibodies (0.1 mg/mL) 24 h prior to aerosol infection with pathogenic M. tb strain HN878. Lung M. tb bacterial burden was determined as CFU at 2 weeks following M. tb infection. Error bars are represented as mean ± SD. In each treatment n = 6 mice. Significance was determined by GraphPad Prism software using Welch’s t-test analysis (p = 0.0215). (TIF) [file ppat.1014133.s005.tif]

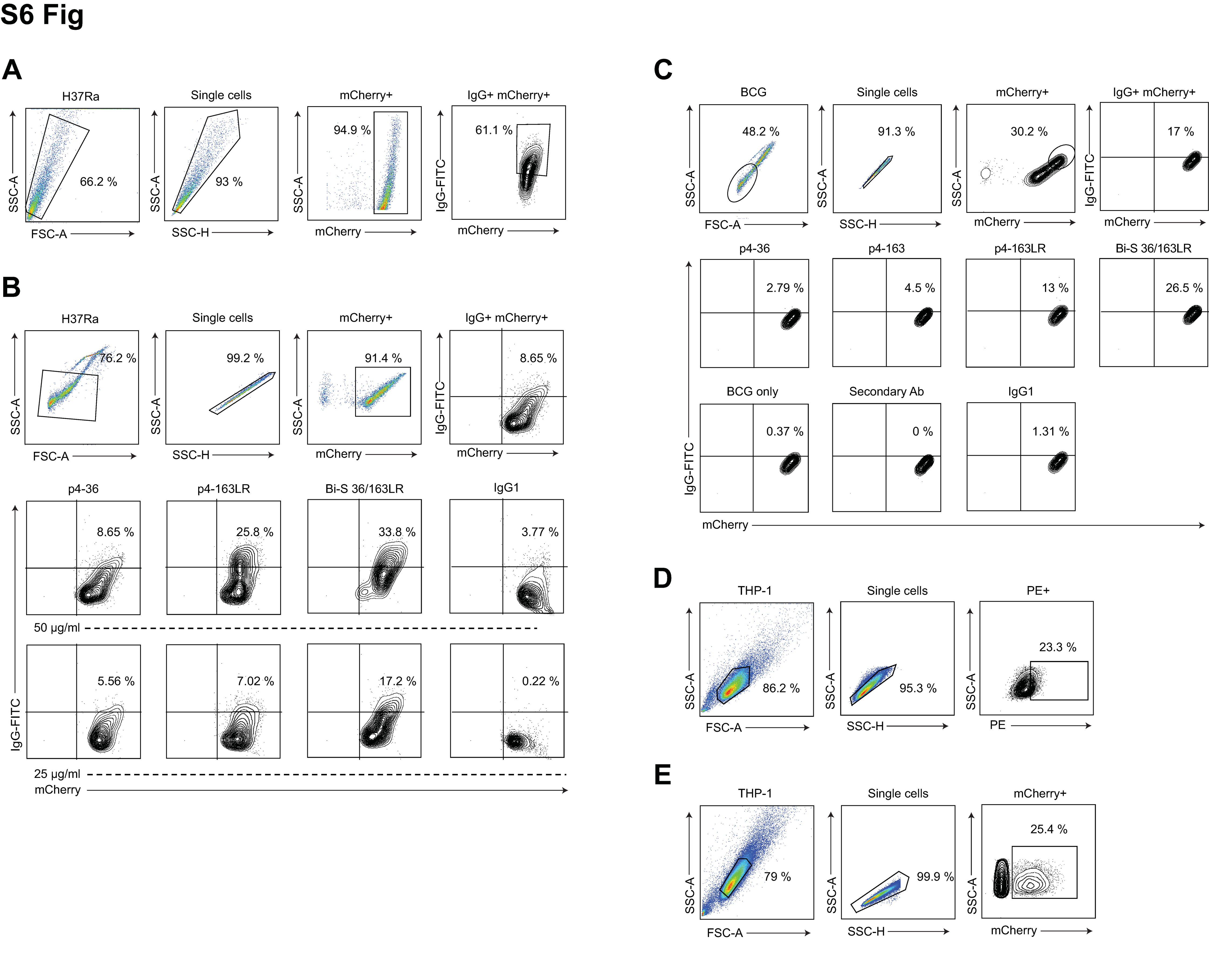

Supplement: S6 Fig — (A) Gating strategy for binding of anti-PstS1 mAbs to live H37Ra-mCherry as determined by flow cytometry. (B) Binding of mAbs to live whole bacteria, H37Ra using two different mAb concentrations, 50 μg/ml and 25μg/ml for p4-36, p4-163LR and Bi-S 36/163LR along with the isotype control. Top panel represents the gating strategy while the lower panels represent the % of IgG-bound bacteria. (C) Gating strategy (top) and flow plots (bottom) for binding of anti-PstS1 mAbs to live BCG-mCherry as determined by flow cytometry. Gating strategy for Antibody-mediated uptake of (D) PstS1-PE and (E) H37Ra-mCherry as determined by flow cytometry of THP-1 monocytic cells. (TIF) [file ppat.1014133.s006.tif]
